# Seroprevalence and associated risk factors for vector-borne pathogens in dogs from Egypt

**DOI:** 10.1186/s13071-021-04670-0

**Published:** 2021-03-22

**Authors:** Abdelfattah Selim, Abdullah D. Alanazi, Alireza Sazmand, Domenico Otranto

**Affiliations:** 1 Department of Animal Medicine (Infectious Diseases), Faculty of Veterinary Medicine, Benha University, Toukh, 13736 Egypt; 2 Department of Biological Sciences, Faculty of Science and Humanities, Shaqra University, P.O. Box 1040, Ad-Dawadimi, 11911 Saudi Arabia; 3 Department of Pathobiology, Faculty of Veterinary Science, Bu-Ali Sina University, Hamedan, 6517658978 Iran; 4 Zoonotic Diseases Research Center, School of Public Health, Shahid Sadoughi University of Medical Sciences, Yazd, 8915173160 Iran; 5 Department of Veterinary Medicine, University of Bari, 70010 Bari, Italy

**Keywords:** *Anaplasma*, *Borrelia*, Canine vector-borne pathogens, *Dirofilaria*, Egypt, *Ehrlichia*, One-health, Zoonosis

## Abstract

**Background:**

Dogs play an important role as reservoirs of many zoonotic vector-borne pathogens worldwide, yet reports of canine vector-borne diseases (CVBDs) in Egypt are scarce.

**Methods:**

Serum samples were collected from pet dogs (*n *= 500) of the three most common breeds (German Shepherd, Rottweiler and Pit Bull) in five Governates of Cairo (*n *= 230), Giza (*n *= 110), Al-Qalyubia (*n *= 60), Al-Gharbia (*n *= 60) and Kafr El-Sheikh (*n *= 40) with a hot desert climate. The presence of antibodies to *Anaplasma* spp. (*A. phagocytophilum*, *A. platys*), *Ehrlichia* spp. (*E. canis*, *E. chaffeensis*, *E. ewingii*), *Borrelia burgdorferi* (*s.l.*) and *Dirofilaria immitis* were assessed using IDEXX SNAP^®^ 4Dx^®^ ELISA tests. For each pathogen, risk factors (i.e. geographical area, keeping condition, sex, age, breed, tick infestation, weekly sanitation of dog enclosures and application of ectoparasiticides) were evaluated by logistic regression approach.

**Results:**

In total, 18.2% (*n *= 91, 95% CI 15.1–21.8) of dogs scored seropositive for at least one pathogen, the most frequent being *Ehrlichia* spp. (*n *= 56; 11.2%; 95% CI 8.7–14.3) followed by *Anaplasma* spp. (*n *= 33; 6.6%, 95% CI 4.7–9.1), *Borrelia burgdorferi* (*s.l.*) (*n *= 9; 1.8%, 95% CI 0.9–3.4) and *D. immitis* (*n *= 7; 1.4%, 95% CI 0.9–2.9). In the tested population, 15.4% (95% CI 12.5–18.8) of dogs were exposed to a single pathogen while 2.4 (95% CI 1.4–4.2) and 0.4% (95% CI 0.1–1.4) were simultaneously exposed to two or three pathogens, respectively. Major risk factors associated with VBDs were living outdoors (*Anaplasma* spp., *P *= 0.0001; *Ehrlichia* spp., *P *= 0.0001), female sex (*Ehrlichia* spp., *P *= 0.005), German Shepherd breed (*Anaplasma* spp., *P *= 0.04; *Ehrlichia* spp., *P *= 0.03), tick infestation (*Anaplasma* spp., *P *= 0.0001; *Ehrlichia* spp., *P *= 0.0001; *B. burgdorferi* (*s.l.*), *P *= 0.003; *D. immitis, P *= 0.02), irregular sanitation (*Anaplasma* spp., *P *= 0.0001; *Ehrlichia* spp., *P *= 0.0001; *B. burgdorferi* (*s.l.*), *P *= 0.002; *D. immitis, P *= 0.01) and not using ectoparasiticides (*Anaplasma* spp., *P *= 0.0001; *Ehrlichia* spp., *P *= 0.0001; *B. burgdorferi* (*s.l.*), *P *= 0.007).

**Conclusion:**

To our knowledge, this is the first large-scale seroepidemiological study of CVBDs in Egypt. Considering that all of the detected pathogens are potentially zoonotic, effective ectoparasite control strategies, regular examination of pet dogs and successful chemoprophylaxis are advocated.

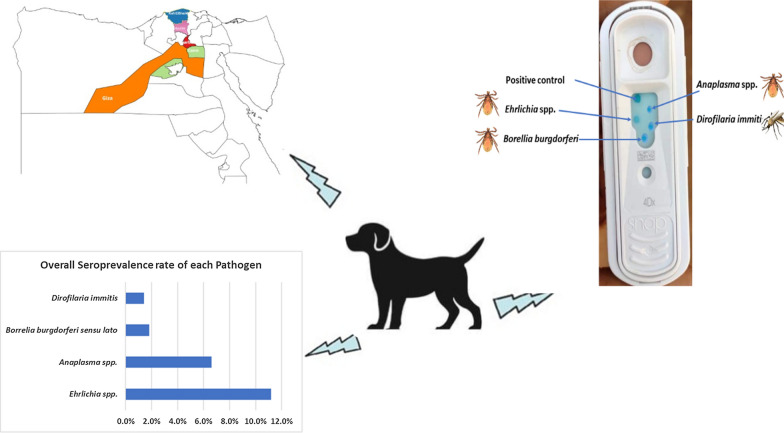

## Background

Vector-borne diseases (VBDs) are of global importance especially in the case of zoonotic infections which pose a direct threat to animal and human health [1–3]. These pathogens are circulated in animal and human communities by arthropod vectors including ticks, mosquitoes, fleas and phlebotominae sand flies [4, 5]. Canine vector-borne diseases (CVBDs) of viral, bacterial and protozoal origin are often widespread in tropical and subtropical regions [6], including in the Middle East and North Africa (MENA), because of the favourable climatic conditions for the perpetuation of arthropod vectors and development of canine vector-borne pathogens (CVBPs) [7]. Among CVBPs, tick-borne *Ehrlichia* spp., *Anaplasma* spp., *Borrelia* spp. and mosquito-borne *Dirofilaria* spp. are of great importance for dogs [8].

Dogs are the main reservoir hosts for zoonotic gram-negative intracellular bacteria *Ehrlichia canis*, *E. ewingii* and *E. chaffeensis* [9, 10]. *Ehrlichia canis*, the causative agent of canine monocytic ehrlichiosis (CME), is transmitted by *Rhipicephalus sanguineus* (*s.l.*) and is prevalent in dog populations worldwide [11, 12]. The clinical outcome of ehrlichiosis varies from mild symptoms to fatal illness in the chronic phase depending on the strain, individual immune response and presence of concomitant infections [4]. Although dogs may show nonspecific signs (e.g., fever, depression, weakness, lethargy, anorexia, weight loss and a mucopurulent nasal discharge), asymptomatic *Ehrlichia* infection may also occur [13–15].

Human and animal infections with *Anaplasma* species are increasingly recognized as important, occasionally emerging and potentially fatal tick-transmitted diseases of humans and animals [16, 17]. Among six recognized species in the genus *Anaplasma*, *A. phagocytophilum*, the causative agent of granulocytic anaplasmosis, is diagnosed in a wide range of warm-blooded hosts including dogs, cats, horses, sheep, goats, cattle, camels and humans [18, 19]. *Anaplasma platys* is a common VBP of dogs in MENA [20, 21] and has occasionally been detected in humans [22–24]. Dogs usually are silent carriers of the infection [25], with clinical signs (e.g. fever, lethargy, anorexia and thrombocytopenia) sometimes described [26].

Among bacteria of the genus *Borrelia*, which affect different animal species including humans, *Borrelia burgdorferi* (*s.l.*) species complex causes Lyme disease, which is considered a major zoonosis for which many animals species (e.g. reptiles, rodents, wild ruminants) are reservoirs and *Ixodes* spp. tick the primary vector [27, 28]. In dogs, *B. burgdorferi* (*s.l.*) most often causes nonspecific signs (e.g., fever, apathy, lethargy, renal damage and lymphadenopathy) but also severe arthritis and neurological disorders [29]. However, in the endemic areas the majority of seropositive dogs do not present any clinical signs of the infection although they often remain persistently infected for approximately 1 year [30].

*Dirofilaria immintis* (Spirurida, Onchocercidae) is the causative agent of canine heartworm disease, which is transmitted through the bite of several mosquito species worldwide [31]. Dirofilariosis may also affect other mammals including humans, leading to the formation of pulmonary nodules, which may be often confounded with pulmonary carcinoma [32]. Although most dogs infected by *D. immitis*—specially in endemic areas—are asymptomatic microfilaremic reservoirs, clinical signs depend on several factors, such as adult worm burden and localization [33]. The distribution of canine dirofilariosis in the MENA region, especially in North Africa, is not well known because of the paucity of epidemiological studies [31].

In Egypt, infection of dogs with *E. canis* [13, 34], *D. immitis* [35] and *B*. *burgdorferi* (*s.l.*) [36, 37] have been reported, in most cases based on small numbers of dogs and limited geographical areas. In this country *Rh. sanguineus* (*s.l.*) (brown dog tick), the competent vector of several tick-borne diseases [38, 39], has been prevalent in dog populations since ancient times [40–43]. DNA of *E. canis* and *A. phagocytophilum* has been detected in ticks attached to dogs [44, 45]. However, generally there are limited data on the occurrence of CVBDs in north African countries, e.g. Morocco [46], Algeria [47, 48] and Tunisia [49, 50].

The aim of the current study was to provide novel information on the seroprevalence and distribution of causative agents of monocytic ehrlichiosis, granulocytic anaplasmosis, Lyme disease and heartworm disease in dogs from five Governorates of Egypt and assess the risk factors associated with the infections.

## Methods

### Study area

**Fig. 1:**
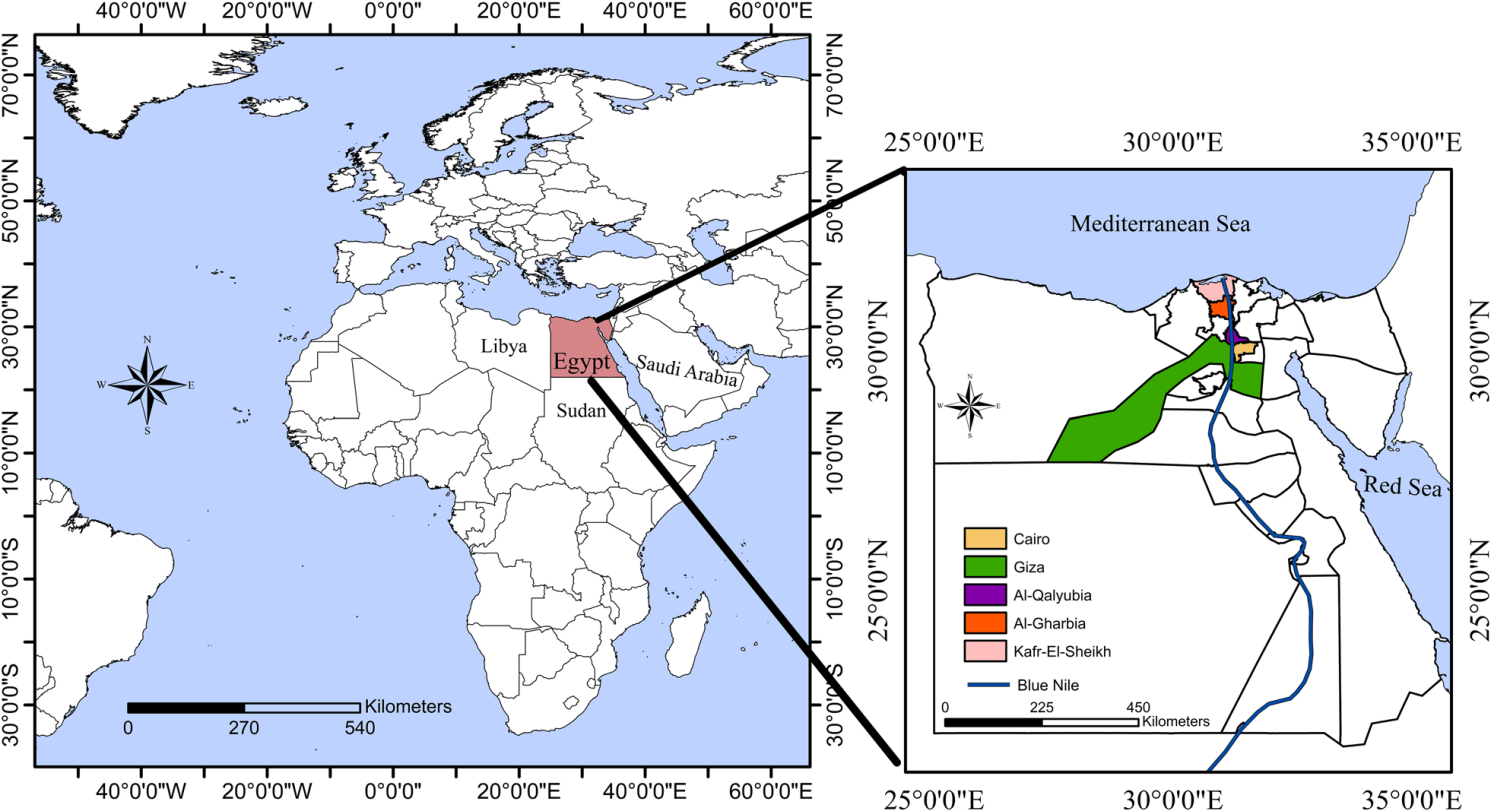
Map indicating Governorates where animals included in the study were sampled

Egypt is a transcontinental country spanning the northeast corner of Africa and southwest corner of Asia. It is divided into 27 Governorates; the large regions of the Sahara desert, which constitute most of Egypt’s territory, are sparsely inhabited. The investigation was conducted in Cairo (30.0444°N, 31.2357°E), Giza (30.0131°N, 31.2089°E), Al-Qalyubia (30.3292°N, 31.2168°E), Al-Gharbia (30.8754°N, 31.0335°E) and Kafr El-Shaikh (31.1107°N, 30.9388°E) (Fig. 1). These Governorates essentially have a hot desert climate, which is classified as BWh by the Köppen-Geiger system.

### Sample collection

During 2018 and 2019, blood samples (ca. 2 ml) were collected from the cephalic or saphenous veins of 500 dogs of the three most common breeds raised in Egypt, i.e. German Shepherd, Rottweiler and Pit Bull, admitted to veterinary clinics of five cities in different Governorates, namely Naser City in Cairo (*n *= 230), 6th of October in Giza (*n *= 110), Benha in Al-Qalyubia (*n *= 60), Tanta in Al-Gharbia (*n *= 60) and Kafr El-Sheikh in the governate of the same name (*n *= 40). Since other breeds of dogs are rarely kept as pets in Egypt, they were excluded from this study. Dogs were grouped according to age into four groups: < 1 year (G1); between 1 and 3 (G2); between 3 and 5 (G3); > 5 years (G4). Animal data (i.e. age, sex, breed, tick infestation, weekly sanitation of the dog enclosures and tri-monthly application of ectoparasiticides) were recorded.

### Serological examination

Sera were separated by centrifugation of blood (1500×*g* for 10 min) and preserved at − 20 °C until tested by IDEXX SNAP^®^ 4Dx^®^ (IDEXX Laboratories, Westbrook, ME, USA), which is a validated in-clinic ELISA test system. The kit simultaneously detects antibodies against immunodominant proteins of *E. canis*, *E. chaffeensis*, *E. ewignii* (peptides from p30 and p30-1 outer membrane proteins and p28 outer surface protein family), *A. phagocytophilum, A. platys* (peptide from the major surface protein p44/MSP2), *B. burgdorferi* (*s.l.*) (C6 peptide, derived from the IR6 region within the *Borrelia* membrane protein VlsE) [51] and *D. immitis* analyte derived from two antibodies (one for capture and the other for detection) specific to heartworm antigens [51–53]. The sensitivity and specificity of this kit are 93.2 and 99.2% for *A. phagocytophilum*, 89.2 and 99.2% for *A. platys*, 96.7 and 98.8% for *B. burgdorferi* (*s.l.*), 97.8 and 92.3% for *E. canis* and 98.9 and 99.3% for *D. immitis* [52–54].

### Statistical analysis

Chi-square test and Fisher’s exact test were used to compare seropositivity to each pathogen, and the results were considered significant if *P *≤ 0.05. In particular, *P*-values were calculated with Fisher’s exact test only for variables below five (keeping condition, sex, weekly sanitation of the dog enclosures, presence of ticks on the dog body and tri-monthly application of ectoparasiticides) for *D. immitis* and *B. burgdorferi* (*s.l.*); all other *P*-values were calculated with Chi-square test. Univariable logistic regression analysis was used to evaluate the association of prevalence of each pathogen and variables of location (five Governorates), keeping condition (indoors or outdoors), sex (male or female), age (three groups), breed (three breeds), weekly sanitation of the dog enclosures, tick infestation and tri-monthly application of ectoparasiticides. Variables with *P *≤ 0.05 in the univariable analyses were conducted to the multivariable models. To determine the risk probability of CBVDs, the odds ratio (OR) and confidence interval (CI) of significant variables were calculated using the multivariant logistic regression model. Multiple linear regression analysis was used to determine the possible multiple collinearities of different variables included in this study. The Hosmer-Lemeshow test was calculated to assess the goodness of fit for each model. Statistical analyses were carried out using SPSS software (ver. 24.0, IBM, USA).

## Results

Of 500 tested dogs, 91 (18.2%) scored seropositive for at least one pathogen, the most frequent being infection with *Ehrlichia* spp. (*n *= 56; 11.2%) followed by *Anaplasma* spp. (*n *= 33; 6.6%), *B. burgdorferi* (*s.l.*) (*n *= 9; 1.8%) and *D. immitis* (*n *= 7; 1.4%). In the tested population 15.4% of dogs were exposed to a single pathogen while 2.4% and 0.4% were simultaneously exposed to two or three pathogens, respectively (Table 1).

**Table 1:** Number and percentage of dogs (*n *= 500) seropositive to *Ehrlichia* spp., *Anapalsma* spp., *B. burgdorferi* (*s.l.*) and *D. immitis*

Pathogen	No. positive	Prevalence (%)	95% CI
Exposure to one pathogen			
*Ehrlichia* spp.	44	8.8	6.5–11.7
*Anapalsma* spp.	24	4.8	3.1–7.1
*B. burgdorferi* (*s.l.*)	5	1	0.3–2.4
*D. immitis*	4	0.8	0.2–2.1
Exposure to two pathogens			
*Ehrlichia* spp. + *Anapalsma* spp.	5	1	0.3–2.4
*Ehrlichia* spp. + *B. burgdorferi* (*s.l.*)	3	0.6	0.2–1.7
*Ehrlichia* spp. + *D. immitis*	2	0.4	0.1–1.4
*D. immitis *+ *Anapalsma* spp.	2	0.4	0.1–1.4
Exposure to three pathogens			
*Ehrlichia* spp. +* Anapalsma* spp. + *D. immitis*	1	0.2	0.04–1.1
*Ehrlichia* spp. +* Anapalsma* spp. + *B. burgdorferi* (*s.l.*)	1	0.2	0.04–1.1

The risk of exposure to pathogens was significantly associated with keeping condition, sex, breed, tick infestation, weekly sanitation of the dog enclosures and tri-monthly application of ectoparasiticides (Table 2). In particular, the risk of infection with *Ehrlichia* spp., *Anaplasma* spp. and *B. burgdorferi* (*s.l.*) was significantly associated with living outdoors, and CME was most prevalent in female dogs. Regarding breed of dogs, German Shepherds showed higher seroprevalence of *Ehrlichia* spp., *Anaplasma* spp. and *B. burgdorferi* (*s.l.*). Importantly, a significantly higher chance of seropositivity to all CVBDs was observed in dogs that lived in enclosures that were not sanitated, did not undergo ectoparasiticide application and were infested with ticks (Table 3). No statistical association was found between CVBDs and other variables. Multicollinearity analysis showed strong correlations between seropositivity to *Anaplasma* spp. and *Ehrlichia* spp. and tick infestation, not receiving adequate hygienic care and ectoparasiticides where the variance inflation factor (VIF) was 15.665 and 25.117, respectively. However, such correlations were observed for seropositivity to *B. burgdorferi* (*s.l.*) and *D. immitis*, i.e. VIF was 1 and 1.082, respectively.

**Table 2:** Risk factors associated with seroprevalence rates of VBDs among dogs in Egypt with single and mixed infection (*n *= 91) according to different variables

Variable	No.	*Ehrlichia* spp.			*Anaplasma* spp.			*Borrelia burgdorferi* (*s.l.*)			*Dirofilaria immitis*		
		Number (%)	95% CI^a^	Chi square	Number (%)	95% CI	Chi square	Number (%)	95% CI	Chi square	Number (%)	95% CI	Chi square
				df^b^			df			df			df
				*P*-value			*P*-value			*P*-value			*P*-value
Governate													
Cairo	230	24 (10.4)	7.1–15	χ^2^ = 6.091 df = 4 *P *= 0.2	15 (6.5)	3.9–10.5	χ^2^ = 5.694 df = 4 *P *= 0.2	5 (2.2)	0.9–4.9	χ^2^ = 2.813 df = 4 *P *= 0.6	4 (1.7)	0.6–4.3	χ^2^ = 1.782 df = 4 *P *= 0.7
Giza	110	19 (17.2)	11.3–25.4		12 (10.9)	6.3–18.1		2 (1.8)	0.5–6.3		2 (1.8)	0.5–6.3	
Al-Qalyubia	60	6 (10)	4.6–20.1		2 (3.3)	0.9–11.3		2 (3.3)	0.9–11.3		1 (1.6)	0.3–8.8	
Al-Gharbia	60	4 (6.6)	2.6–15.9		3 (5)	1.7–13.7		0	0–6		0	0–6	
Kafr El-Sheikh	40	3 (7.5)	2.5–19.8		1 (2.5)	0.4–12.8		0	0–8.7		0	0–8.7	
Keeping condition													
Indoor	310	12 (3.8)	2.2–6.6	χ^2^ = 44.060 df = 1 *P *= **0.0001**	9 (2.9)	1.5–5.4	χ^2^ = 18.086 df = 1 *P *= **0.0001**	2 (0.6)	0.2–2.3	χ^2^ = 6.155 df = 1 *P *= **0.03**	2 (0.6)	0.1–2.3	χ^2^ = 2.071 df = 1 *P *= 0.1
Outdoor	190	44 (23.2)	17.7–29.6		24 (12.6)	8.6–18.1		7 (3.6)	1.7–7.4		5 (2.6)	1.1–6.1	
Sex													
Male	230	16 (6.9)	4.1–11	χ^2^ = 7.712 df = 1 *P *= **0.005**	11 (4.7)	2.6–8.3	χ^2^ = 1.525 df = 1 *P *= 0.6	2 (0.8)	0.2–3.1	χ^2^ = 2.086 df = 1 *P *= 0.2	2(0.8)	0.2–3.1	χ^2^ = 0.868 df = 1 *P *= 0.3
Female	270	40 (14.8)	11.1–19.5		22 (8.2)	5.4–12		7 (2.5)	1.2–5.2		5 (1.8)	0.7–4.2	
Age													
< 1 year	30	3 (10)	3.4–25.6	χ^2^ = 7.525 df = 3 *P *= 0.06	2 (6.6)	1.8–21.3	χ^2^ = 2.1.525 df = 3 *P *= 0.6	1(3.3.)	0.5–16.6	χ^2^ = 2.992 df = 3 *P *= 0.4	0	0–11.3	χ^2^ = 1.525 df = 3 *P *= 0.6
1–3 years	210	16 (7.6)	4.7–12		11 (5.2)	2.9–9.1		2 (0.9)	0.2–3.4		2 (0.9)	0.2–3.4	
3–5 years	180	22 (12.2)	8.2–17.8		15 (8.3)	5.1–13.3		3 (1.6)	0.5–4.7		3 (1.6)	0.5–4.7	
> 5 years	80	15 (18.7)	11.7–28.6		5 (6.2)	2.7–13.8		3 (3.7)	1.2–10.4		2 (2.5)	0.6–8.6	
Breed													
German Shepherd	260	38 (14.6)	10.8–19.4	χ^2^ = 6.870 df = 2 *P *= **0.03**	24 (9.2)	6.2–13.3	χ^2^ = 6.290 df = 2 *P *= **0.04**	6 (2.3)	1.1–4.9	χ^2^ = 1.161 df = 2 *P *= 0.5	4 (1.5)	0.6–3.8	χ2 = 0.550 df = 2 *P *= 0.7
Rottweiler	110	10 (9.1)	5–15.9		5 (4.5)	1.9–10.2		2 (1.8)	0.5–6.3		2 (1.8)	0.5–6.3	
Pit Bull	130	8 (6.2)	3.1–11.6		4 (3.1)	1.2–7.6		1 (0.7)	0.1–4.2		1 (0.7)	0.1–4.2	
Season													
Spring	190	26 (13.6)	9.5–19.3	χ^2^ = 4.416 df = 3 *P *= 0.2	12 (6.3)	3.6–10.7	χ^2^ = 3.938 df = 3 *P *= 0.3	4 (2.1)	0.8–5.2	χ^2^ = 1.400 df = 3 *P *= 0.7	3 (2.1)	0.8–5.2	χ^2^ = 2.955 df = 3 *P *= 0.4
Summer	175	21 (12)	7.9–17.6		16 (9.1)	5.7–14.3		4 (2.2)	0.8–5.7		4 (2.2)	0.8–5.7	
Autumn	90	7 (7.7)	3.8–15.2		4 (4.4)	1.7–10.8		1 (1.1)	0.2–6		0	0.0–5.1	
Winter	45	2 (4.4)	1.2–14.8		1 (2.2)	0.3–11.5		0	0.0–9.8		0	0.0–9.8	
Weekly sanitation of the dog enclosures													
Yes	370	23 (6.2)	4.2–9.2	χ^2^ = 35.346 df = 1 *P *= **0.0001**	10 (2.7)	1.4–4.9	χ^2^ = 35.064 df = 1 *P *= **0.0001**	2 (0.5)	0.1–1.9	χ^2^ = 12.771 df = 1 *P *= **0.002**	2 (0.5)	0.1–1.9	χ^2^ = 7.615 df = 1 *P *= **0.02**
No	130	33(25.3)	18.6–33.4		23 (18.5)	12.7–26		7 (5.4)	2.6–10.6		5 (3.8)	1.6–8.6	
Presence of tick on the dog body													
Yes	140	48 (34.3)	26.9–42.5	χ^2^ = 104.196 df = 1 *P *= **0.0001**	22 (15.7)	10.6–22.6	χ^2^ = 26.203 df = 1 *P *= **0.0001**	7(5)	2.4–9.9	χ^2^ = 11.264 df = 1 *P *= **0.003**	5 (3.5)	1.5–8.1	χ^2^ = 6.642 df = 1 *P *= **0.02**
No	360	8 (2.2)	1.1–4.3		11 (3.1)	1.7–5.3		2 (0.5)	0.12		2 (0.5)	0.1–2	
Tri-monthly application of ectoparasiticides													
Yes	370	24 (6.4)	4.4–9.4	χ^2^ = 31.790 df = 1 *P *= **0.0001**	11 (2.9)	1.6–5.2	χ^2^ = 30.370 df = 1 *P *= **0.0001**	2 (0.5)	0.1–1.9	χ^2^ = 8.604 df = 1 *P *= **0.007**	2 (0.5)	0.1–1.9	χ^2^ = 7.615 df = 1 *P *= **0.02**
No	130	32 (24.6)	18–32.6		22 (16.9)	11.4–24.2		7 (5.4)	2.6–10.6		5 (3.8)	1.6–8.6	
Total	500	56 (11.2)	8.7–14.3		33 (6.6)	4.7–9.1		9 (1.8)	0.9–3.3		7 (1.4)	0.9–2.9	

*P*-values were calculated with Fisher’s exact test only for variables below five (keeping condition, sex, weekly sanitation of the dog enclosures, presence of tick on the dog body and tri-monthly application of ectoparasiticides) for *D. immitis* and *B. burgdorferi* (*s.l.*); all other *P*-values were calculated with Chi-square test

Significant variables (*P*-values ≤ 0.05) are marked in bold

^a^Confidence interval

^b^Degrees of freedom

**Table 3:** Multivariant logistic regression analysis of risk factors associated with seroprevalence rates of VBDs in dogs in Egypt with single and mixed infection (*n *= 91) according to different variables

Pathogen	Factor	ß^a^	SE^b^	Odds ratio	95% Confidence interval	*P*
*Ehrlichia* spp.	Keeping condition					
	Outdoor	2.013	0.314	7.5	3.8–14.6	0.0001*
	Sex					
	Female	0.844	0.311	2.3	1.2–4.2	0.007⃰
	Breed					
	Pit Bull (constant)	–	–	–	–	–
	German Shepherd	0.959	0.405	2.6	1.2–5.7	0.02⃰
	Rottweiler	0.422	0.493	1.5	0.6–4	0.3
	Weekly sanitation of the dog enclosures					
	No	1.636	0.295	5.1	2.8–9.1	0.0001⃰
	Presence of tick on the dog body					
	Yes	3.143	0.399	22.9	10.4–50.2	0.0001⃰
	Tri-monthly application of ectoparasiticides					
	No	1.549	0.293	4.7	2.6–8.4	0.0001⃰
*Anaplasma* spp.	Keeping condition					
	Outdoor	1.576	0.403	4.8	2.1–10.6	0.001⃰
	Breed					
	German Shepherd	1.117	0.553	3.1	1.1–9.1	0.04⃰
	Rottweiler	0.405	0.684	1.5	0.4–5.7	0.5
	Weekly sanitation of the dog enclosures					
	No	2.046	0.394	7.7	3.5–16.7	0.0001⃰
	Presence of tick on the dog body					
	Yes	1.778	0.384	5.9	2.7–12.5	0.0001⃰
	Tri-monthly application of ectoparasiticides					
	No	1.894	0.385	6.6	3.1–14.1	0.0001⃰
*B. burgdorferi* (*s.l.*)	Keeping condition					
	Outdoor	1.773	0.807	5.8	1.2–28.6	0.02⃰
	Weekly sanitation of the dog enclosures					
	No	2.349	0.809	10.5	2.1–51.1	0.004⃰
	Presence of tick on the dog body					
	Yes	2.243	0.808	9.4	1.9–45.9	0.006⃰
	Tri-monthly application of ectoparasiticides					
	No	2.439	0.809	10.5	2.1–51.1	0.004⃰
*D. immitis*	Weekly sanitation of the dog enclosures					
	No	1.966	0.843	7.4	1.4–38.4	0.02⃰
	Presence of tick on the dog body					
	Yes	1.673	0.843	6.6	1.3–34.5	0.02⃰
	Tri-monthly application of ectoparasiticides					
	No	1.966	0.843	7.4	1.4–38.4	0.02⃰

ß: Wald statistic; SE: standard error; 95% CI: 95% confidence interval; OR: odds ratio

*These parameters are statistically significant

## Discussion

Data presented indicate that dog populations (i.e. 18.2%) in Egypt are exposed to CVBP, therefore posing threats to their own health and to people. This is largely due to the wide distribution of *Rh. sanguineus* (*s.l.*), the most common tick species infesting dogs in Egypt and a vector of canine ehrlichiosis and anaplasmosis [40–42]. Though little information is available about the prevalence of CVBDs in the MENA region, dogs often act as reservoirs of VBPs with prevalence of 18.8% in Qatar [55], 24.5% in Saudi Arabia [24], 38.1% in Iraq [21], 46.9% in Iran [20], 69.7% in Algeria [48], 73% in Israel [56] and 83.8% in Morocco [46]. In Egypt CVBDs were mostly observed in urban Governorates (i.e. Giza, Cairo and Al-Qalyubia) where keeping pet animals is more common, also indicating that dogs and people in these regions are at higher risk of acquiring VBDs of canine origin.

The most prevalent pathogen diagnosed in this study was *Ehrlichia* spp. An *E. canis* seroprevalence of 41% was reported in dogs from Cairo and Alexandria [34] where DNA of *E. canis* was also detected in *Rh. sanguineus* (*s.l.*) ticks collected on dogs [45]. In addition, the presence of *E. canis* is supported by the wide distribution in Egypt of the brown dog tick *R. sanguineus* (*s.l.*) [40], which is the recognized vector for this species [57]. Although the employed test could detect exposure to *E. canis*, *E. chaffeensis* and *E. ewingii*, in a previous study only *E. canis*, but not *E. ewingii* or *E. chaffeensis*, was molecularly diagnosed in blood of 39 seropositive dogs from Cairo, Giza and Al-Qalyubia [58]. Nonetheless, since *E. ewingii* and *E. chaffeensis* have been diagnosed in dogs, ticks and human patients from different countries in the African continent, e.g. Cameroon, Mali, Uganda and South Africa [59–61], further investigations should be carried out to characterize the species of *Ehrlichia* infecting dogs in Egypt.

To the best of our knowledge this is the first report of sero-reaction to *A. phagocytophilum*/*A. platys* in dogs from Egypt (6.6%). It seems that *A*. *phagocytophilum* infection of dogs is not prevalent in some regions of MENA, also considering that previous studies from Saudi Arabia, Qatar, Iraq and five regions of Iran failed to detect the infection [20, 21, 55, 62]. However, both *A. phagocytophilum* and *A. platys* were detected in *Rh. sanguineus* (*s.l.*) blood-feeding on dogs in Cairo and Giza [44, 45] and *A. phagocytophilum* in blood of five human patients in the Nile Delta [63].

The detection of nine dogs (1.8%) seropositive to *B. burgdorferi* (*s.l.*) suggests that the pathogen circulates in Egypt as indicated by previous studies where prevalence ranged from 23% [36] to 71.4% [37]. In addition, some tick species, e.g. *Rh. sanguineus* (*s.l.*), *Rh. annulatus*, *Hyalomma excavatum*, *Hy. dromedarii*, *Amblyomma lepidum* and *Ornithodoros savignyi*, scored molecularly positive for DNA of *B. burgdorferi* (*s.l.*) [36, 64]. The circulation of *B. burgdorferi* (*s.l.*) in Egypt has also been demonstrated [36, 63, 65, 66]. In addition, tick-borne relapsing fever (TBRF) caused by *Borrelia persica*, *Borrelia microti*, *Borrelia latyschewii* and *Borrelia baltazardi* is also endemic in MENA [67] including Egypt where relapsing fever *Borrelia* spp. were detected in *Ornithodoros savignyi* ticks and sera of camel, sheep, goat, cattle and buffalo [68, 69]. In particular, *B. persica*, which is transmitted by *Ornithodoros tholozani*, can infect dogs and has been reported from Egypt, Israel, Iran, Pakistan and former USSR Asian republics including Uzbekistan [70, 71]. Considering that yet available commercial point-of-care diagnostic kits such as SNAP^®^ 4Dx^®^ employed in this study do not detect TBRF in dogs, complementary tests for dogs living in the endemic areas are recommended.

The detection of seven dogs (1.4%) from Giza, Cairo and Al-Qalyubia seropositive to *D. immitis* corroborates an older report of microfilariae of *D. immitis* in blood smears from 8/19 imported German Shepherd dogs in Assiut Governorate, Upper Egypt [35]. In particular, antibodies to *D. immitis* were already detected from cats of Giza with prevalence of 3.4% [72]. While human cases with *D. repens* have been frequently reported in Egypt [73, 74], *D. immitis* was described once in a patient [75]. Data for canine dirofilariosis by *D. immitis* in North Africa are limited to few reports from Tunisia [49, 76], Algeria [77, 78] and Morocco [46]. It has been suspected that *D. immitis* is absent from some Middle Eastern countries such as Israel where *D. repens* is present [79]. In contrast, weighted prevalence of *D. immitis* infection in dog populations of Turkey and Iran was estimated to be 11.32% and 11.45%, respectively [80]. However, in a recent study in five geographical regions of Iran testing 354 dogs, no microfilaremic dog was found [20].

According to our findings, dogs that were living outdoors had a higher risk of being seropositive to *Ehrlichia* spp., *Anaplasma* spp. and *B. burgdorferi* (*s.l.*) probably as an effect of the higher chances of being exposed to bites of the brown dog tick *Rh. sanguineus* (*s.l.*) and other tick species that are competent vectors of these pathogens [5]. Furthermore, as expected, dogs that did not receive adequate hygienic care and antiparasitic treatments were more likely to be affected by CVBPs. Regular application of ectoparasiticides with potent anti-feeding and fast killing effects, repellents, insect growth regulators and juvenile hormone analogues combined with environmental treatment to reduce the number of adult and juvenile ticks are key control measures in managing CVBDs [81]. Ectoparasiticides, available in several formulations, such as pour-on, spot-on, shampoos, sprays and collars, have long-lasting effects [82] and are highly recommended to dog owners to prevent CVBDs.

In this study, seropositivity to *Ehrlichia* spp. was more frequent in female dogs. In contrast, some studies have found higher seropositivity to CVBDs in males due to behavioural characteristics that cause greater exposure to vectors than females [83, 84]. No sex-related correlation was recorded for other tested pathogens in this study.

German Shepherds showed higher seroprevalences of *Ehrlichia* spp. and *Anaplasma* spp. Although all breeds are prone to CVBDs, CME has been reported most frequently in the German Shepherds [85, 86]. German Shepherd dogs and Siberian Huskies are predisposed to developing more severe clinical signs of ehrlichiosis; therefore, these breeds have a worse prognosis [14]. An experimental study showed that the cell-mediated immune response to a challenge with *E. canis* was reduced in German Shepherds compared to Beagle dogs [87]. Examination of other dog breeds in Egypt though are very rare, and “*baladi*” stray dogs will shed light on the true occurrence of CVBDs in the country.

As a limitation of the employed commercial kit, PCR-positive/antibody-negative and antibody-positive/PCR-negative dogs have been reported [88]. The first case could represent early infections before the development of antibody responses; the second case may represent cases of past infections that may have been treated or spontaneously resolved. Furthermore, this commercial kit detects both IgM and IgG antibodies against *Erhlichia* spp., *Anaplasma* spp. and *B. burgdorferi* (*s.l.*). Although chronic long-term bacteremia is characteristic for some rickettsial agents, long-term persistence of IgG could occur, not reflecting the real “infection” status in some animals. Hence, seropositivity values must be interpreted as current infection with or previous exposure to the pathogens under assessment. Finally, the prevalence of *D. immitis* infection in Egypt should be carefully considered as an alarm bell for the introduction of a parasite not common in Egypt but which is expanding its range of distribution in southern regions of the Mediterranean Basin such as southern Italy [89].

## Conclusion

The present study is the first comprehensive study for CVBD pathogens that has been conducted in Egypt to our knowledge and confirms the presence of *Ehrlichia* spp., *Anaplasma* spp., *B. burgedorferi* (*s.l.*) and *D. immitis* in dogs of different regions. The connection between these VBPs and their arthropod vectors in Egypt remains largely unknown and warrants further investigation. Considering that all of the detected pathogens are known zoonotic pathogens, effective ectoparasite control strategies, regular examination of pet dogs and successful chemoprophylaxis are advocated.

## Data Availability

All data generated or analysed during this study are included in this published article and its additional files.

## Abbreviations

CVBPs: Canine vector-borne pathogens
CVBDs: Canine vector-borne diseases
OR: Odds ratio
MENA: Middle East and North Africa
DNA: Deoxyribonucleic acid
CME: Canine monocytic ehrlichiosis
TBRF: Tick-borne relapsing fever
CI: Confidence interval
ELISA: Enzyme-linked immunosorbent assay
s.l.: Sensu lato
VIP: Variance inflation factor
IR6: Invariable region 6
VlsE: Vmp-like sequence
df: Degrees of freedom
